# Reframing the substrate as an active process component in fungal solid-state fermentation of foods

**DOI:** 10.1038/s41538-026-00827-2

**Published:** 2026-04-04

**Authors:** Simon Müller, Carole Zermatten, Till Germerdonk, Patrick A. Rühs

**Affiliations:** Institute of Food, Nutrition and Health, ETH Zürich, Zürich, Switzerland

**Keywords:** Biochemistry, Biotechnology, Engineering, Microbiology

## Abstract

Fungal solid-state fermentation can improve the nutritional quality of plant-based foods, yet most processes rely on substrates whose properties are inherited from raw materials rather than intentionally designed. This perspective addresses this limitation by reframing the substrate as an active process component. Integrating biochemical, mechanical, and architectural principles into substrate design enables the development of engineered substrates that promote volumetric and spatially uniform bioconversion during solid-state fermentation of food materials.

## Introduction

Plant-based foods are increasingly recognized for their environmental benefits, yet their protein digestibility and mineral bioavailability are often limited due to restricted enzymatic accessibility and the presence of anti-nutritional factors (ANFs)^1,2^. Improving the nutritional quality of plant raw materials is therefore a key challenge for transitioning to sustainable, health-promoting food systems.

Fungal solid-state fermentation (SSF) offers a biotechnological route to transform plant materials into foods with improved nutritional quality^3^. Filamentous fungi can improve protein digestibility, balance amino acid profiles, degrade ANFs, and generate beneficial secondary metabolites^4^. SSF has long been used in traditional fermented foods such as tempeh, miso, and koji, and is increasingly applied in modern bioprocessing to valorize plant proteins and side-stream materials^5–7^.

To date, research has focused largely on optimizing fungal metabolism, substrate formulation, and process engineering aspects in SSF. These efforts have advanced understanding of how the enzymatic repertoires of different strains influence the nutritional value of specific substrates^5^, and how diverse raw materials can serve as substrates for SSF^8,9^. In parallel, process engineering strategies, such as aeration control and bioreactor design, have improved mass and heat transfer during fermentation^6,10,11^.

Despite these advances in fungal biology and process engineering, the substrate’s physical properties have remained largely unchanged. Most SSF processes continue to rely on particulate substrates such as grains, legumes, or their processing fractions, whose biological origin determines their structure and mechanical properties. These particulate materials limit oxygen diffusion, restrict nutrient accessibility, and have fixed mechanical properties^11,12^. As a result, fungal growth and enzymatic activity are restricted to surface layers, leading to spatially heterogeneous bioconversion. While bioreactor innovations and aeration strategies can mitigate these limitations, they do not address the core issue: the substrate remains a passive medium rather than a designed component of the process. This reliance on conventional particulate substrates is economically practical and compatible with existing infrastructure, which partly explains its persistence in SSF processes. At the same time, the continued emphasis on process interventions shows that transport limitations and heterogeneous colonization remain technically and economically relevant challenges. Addressing these constraints at the substrate level, therefore, represents a complementary strategy to improve substrate utilization and enable more uniform bioconversion throughout the material.

**Fig. 1:**
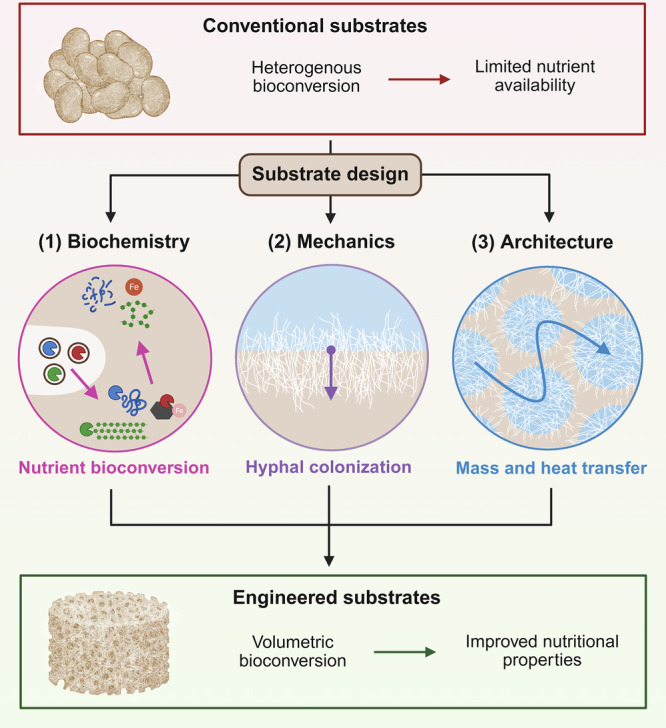
Substrate design framework for fungal solid-state fermentation of foods. Conventional particulate substrates have fixed biochemical composition, mechanical properties, and architecture, resulting in heterogeneous bioconversion and limited nutrient availability. Treating the substrate as a designable process component introduces three substrate design principles: (1) biochemical affinity between strain and substrate shapes nutrient bioconversion; (2) substrate mechanics regulate hyphal colonization; and (3) substrate architecture governs mass and heat transfer. Intentional design of these properties enables the development of engineered substrates that support volumetric bioconversion and improved nutritional properties. Figure was created with BioRender.

In this perspective, we propose three design principles that redefine the substrate as an engineered component of the SSF process (Fig. 1): (1) biochemical affinity between strain and substrate shapes nutrient bioconversion; (2) substrate mechanics regulate hyphal colonization; and (3) substrate architecture governs mass and heat transfer and enables volumetric growth. Together, these principles guide the design of engineered substrates that promote volumetric and spatially uniform bioconversion in plant-based foods.

## (1) Biochemical affinity between strain and substrate shapes nutrient bioconversion

**Fig. 2:**
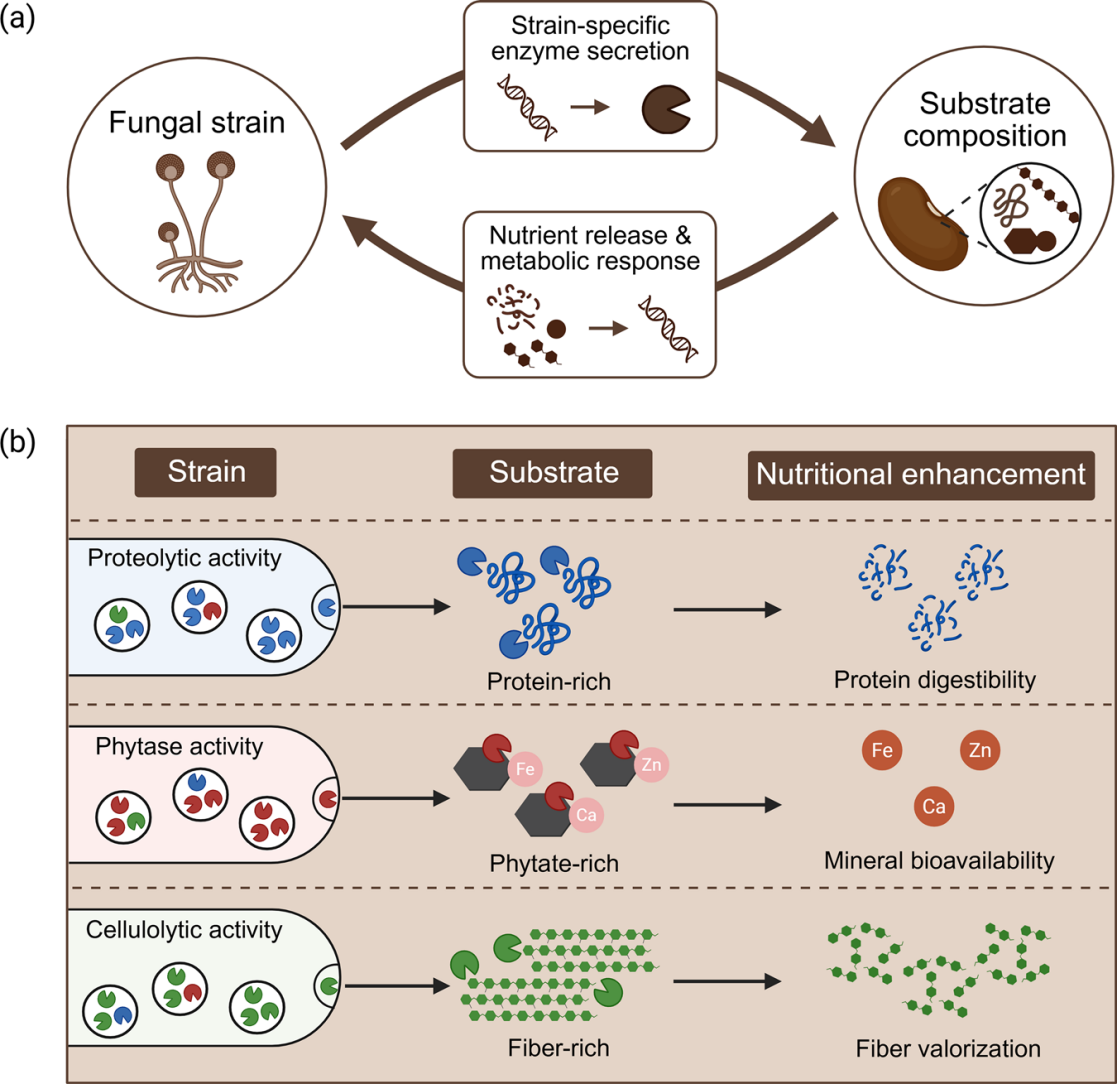
Strain-substrate affinity shapes nutrient bioconversion in fungal solid-state fermentation. **a** Fungal strains secrete strain-specific extracellular enzymes that degrade substrate components, releasing nutrients that trigger metabolic responses and feedback on enzyme production. **b** Distinct enzymatic repertoires of fungal strains interact with substrate composition to achieve targeted nutrient transformations. Proteolytic strains acting on protein-rich substrates improve protein digestibility, phytase-active strains on phytate-rich substrates enhance mineral bioavailability, and cellulolytic strains on fiber-rich substrates enable fiber valorization. Figure was created with BioRender.

Biochemical affinity between a fungal strain and substrate composition governs the efficiency and specificity of nutrient bioconversion in SSF (Fig. 2)^2^. Filamentous fungi acquire nutrients through absorptive heterotrophy. They secrete extracellular enzymes that break down complex biomolecules into soluble compounds^13^. The absorbed nutrients fuel their metabolism for biomass formation and secondary metabolite production. Changes in nutrient availability are sensed by the fungus and trigger shifts in gene expression and enzyme secretion, creating a feedback loop between substrate composition and fungal metabolic responses (Fig. 2a)^14^. This bidirectional interaction allows fungi to exploit diverse plant materials and agricultural side streams as a nutrient source^5,13,15^. Three groups of enzymatic activities are central to improving the nutritional quality of plant substrates: proteolytic, phytase, and cellulolytic activity.

### Enzymatic bioconversion

Proteolytic enzymes hydrolyze proteins into short peptides or free amino acids, improving their digestibility (Fig. 2b)^2,16^. Fungal metabolism also affects proteins through amino acid recycling: fungi assimilate amino acids from the substrate and re-assemble them into fungal proteins^17^. The resulting mycoproteins often display a more balanced amino acid profile, improving the digestible indispensable amino acid score (DIAAS) and compensating for limiting amino acids in plant-based diets^17,18^. Mineral bioavailability in plant raw materials is often limited by ANFs such as phytates, tannins, oxalates, and lectins that form insoluble complexes with minerals or bind to the gut epithelium, reducing absorption efficiency^19,20^. Mineral bioavailability can be enhanced through the hydrolysis of those ANFs by fungal enzymes^2^. For instance, phytases degrade phytates and liberate the bound iron, zinc, and calcium ions, improving their bioavailability (Fig. 2b)^21^. Carbohydrate-active enzymes like cellulase and laccase break down complex polysaccharides, fibers, and lignin-associated components, releasing nutrients that would otherwise remain inaccessible (Fig. 2b)^15^. This enzymatic activity improves the digestibility of fiber-rich plant materials, such as agricultural side streams. Moreover, many plant raw materials contain oligosaccharides such as raffinose and stachyose that humans cannot enzymatically digest. These compounds are instead fermented by the gut microbiota, often resulting in gas production. Oligosaccharidases secreted by filamentous fungi hydrolyze these undesirable components and reduce digestive discomfort^22,23^.

### Strain-substrate affinity

Fungal strains possess distinct enzymatic repertoires shaped by their genetic makeup and ecological specialization^24^. For example, proteolytic strains thrive on high protein, cellulolytic strains on high fiber, and strains with phytase activity on phytate-rich substrates (Fig. 2)^15^. Nutrient availability further regulates gene expression and enzyme secretion, enabling a strain to adapt its enzymatic profile to different substrates. This diversity in enzyme secretion suggests a broad potential for selecting fungal strains to match defined bioconversion objectives^13,25^. Empirical strain-substrate matching is evident in established SSF food products. In tempeh production, *Rhizopus oligosporus* is cultivated on soybeans, a protein-rich substrate compatible with its proteolytic activity. In koji fermentation, *Aspergillus oryzae* is cultivated on rice or cereals, where its amylolytic enzymes hydrolyze starch-rich materials. These examples demonstrate that biochemical matching between strain and substrates is possible in practice. However, such pairings emerged through historical practice rather than systematic design^26^. Despite extensive understanding of fungal metabolism, modern SSF food processes rarely treat strain selection and substrate composition as intentional design parameters^26–28^. Substrates are typically derived from traditional raw materials and used with minimal modification^29^, while strain selection often reflects historical precedent rather than mechanistic matching^30,31^. In food applications, strain selection is further constrained by regulatory approval (GRAS status), which limits the range of organisms available for pairing with a given substrate. Because fermentation outcomes are highly strain specific, nutritional improvements such as protein digestibility, amino acid balance, and ANF reduction vary widely^2^. Systematic strain-substrate pairing remains limited because interactions in solid substrates are difficult to characterize, and conventional analytical methods often require destructive or extraction-intensive sample preparation^5^. Moving strain-substrate pairing from an empirical practice to design-driven fermentation will require improved characterization of solid substrates, enzyme activities, and fungal metabolic responses.

### Toward systematic strain-substrate selection

Strain-substrate pairings must become more systematic. High-throughput screening methods could generate standardized datasets describing enzyme activities, substrate composition, and bioconversion outcomes. Integrating such datasets into strain-substrate interaction databases would provide a foundation for predictive modeling. Modeling approaches, including machine learning, may then support the identification of strain-substrate combinations for defined bioconversion objectives. Establishing such a data infrastructure would move SSF from empirical pairing toward data-driven strain selection.

## (2) Substrate mechanics regulate hyphal colonization

**Fig. 3:**
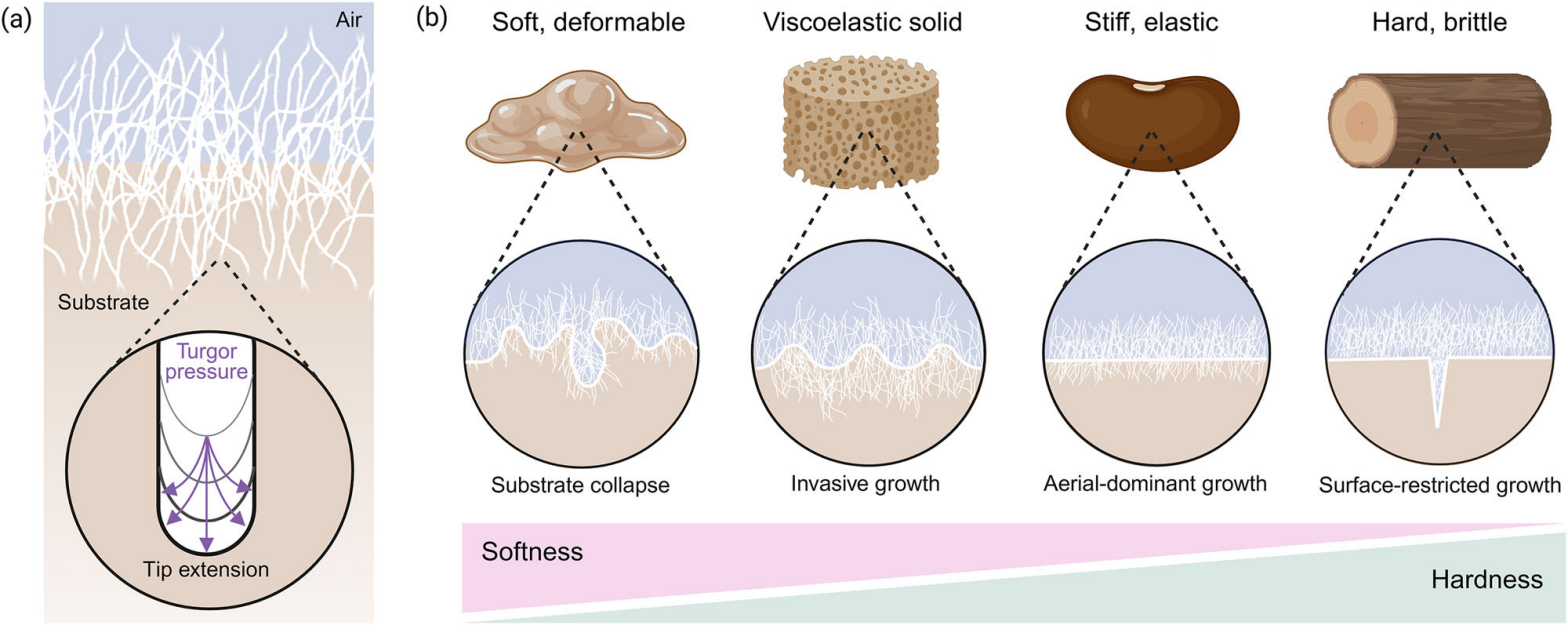
Substrate mechanics regulate fungal colonization patterns in solid-state fermentation. **a** Fungal hyphae extend by generating turgor pressure at the growing tip, resulting in penetration or surface growth depending on the mechanical resistance of the substrate. **b** Substrates with different mechanical properties result in distinct colonization modes. Soft, deformable substrates may collapse under growth conditions, whereas viscoelastic solids support invasive hyphal growth while maintaining structural integrity. Increasing stiffness and elasticity favor predominantly aerial growth, while hard and brittle substrates resist penetration, resulting in surface-restricted colonization. Figure was created with BioRender.

The mechanical properties of the substrate determine how fungi attach, penetrate, and spread during SSF, directly influencing the depth of colonization and nutrient bioconversion. Fungal hyphae grow by generating internal turgor pressure that drives tip extension against the surrounding material (Fig. 3a)^32^. At the growing tip, localized remodeling of the cell wall allows turgor pressure to expand the wall, while new polysaccharides are inserted to support continuous growth^33^. Whether these forces result in invasive or surface-restricted growth depends on the substrate’s mechanical resistance to deformation. A given fungal strain has a stiffness threshold above which hyphal penetration is strongly reduced, reflecting the maximum pressure that the hyphal tip can exert^34^. Above this threshold, fungi shift to surface growth and rely on enzymatic degradation to soften the substrate^35^. Despite this clear coupling between substrate mechanics and fungal colonization, mechanical properties remain poorly quantified and are rarely designed intentionally in SSF^36–38^.

### Colonization across different substrate mechanics

Fungal colonization depends on the balance between hyphal forces and the mechanical resistance of the substrate. This balance determines whether growth is invasive, surface-restricted, or enabled over time through enzymatic softening (Fig. 3b). In very soft and low-yield stress substrates such as slurries or low-concentration hydrogels, hyphae encounter minimal mechanical resistance. This allows formation of penetrative hyphae, but with limited structural support from the substrate^36^. Collapse or fluidization of the substrate reduces its structural integrity and allows hyphae to grow within a mechanically unconfined matrix^39^. In addition to bulk mechanical resistance, interfacial properties influence early hyphal attachment and surface growth. Filamentous fungi secrete hydrophobins, which are small, amphipathic surface proteins that lower interfacial tension and promote adhesion at air-substrate interfaces^40–42^. These proteins facilitate attachment and aerial hyphae formation, particularly on soft or weakly structured materials, but do not reduce the mechanical force required for substrate penetration, which remains governed by bulk mechanics. Viscoelastic solid materials, including concentrated hydrogels, solid foams, and porous biopolymer scaffolds, deform locally while retaining their macroscopic structure^37,43^. This allows hyphal penetration while maintaining substrate integrity. Hydrogel substrates based on different polysaccharides or proteins have been shown to impact radial extension rate and penetrative growth: higher network strength generally promotes faster radial extension and denser surface growth, whereas softer gels support deeper penetration into the substrate^37,44^. Stiff and predominantly elastic substrates such as high-concentration agar gels or cooked legumes, resist deformation under hyphal pressure^38^. Growth is largely confined to the air-substrate interface, forming dense aerial mycelium but limited internal colonization^38^. Hard and brittle materials, including dry lignocellulosic substrates such as wood, bran particles, or untreated plant fibers, present mechanical barriers that hyphae cannot overcome directly^45^. Hyphae primarily grow across the surface or within pre-existing pores or cracks^46^. Internal colonization depends on gradual enzymatic softening that enables deeper colonization over time. Early growth is therefore restricted to the surface, resulting in slow and spatially heterogeneous bioconversion^47^. A comparable relationship between substrate mechanics and colonization is evident in legume-based SSF systems such as tempeh. Soybeans are soaked and thermally treated prior to fermentation, which softens the cotyledon structure and facilitates hyphal penetration by *R. oligosporus* into internal soybean tissues.

### Effects of mechanics on enzymatic diffusion

Mechanical properties influence not only hyphal colonization, but also the movement of secreted enzymes within the substrate. Because enzymes move mainly through aqueous domains, their transport depends on the presence of water-filled pores, water activity, and substrate microstructure^48,49^. As water activity and the size of water-filled pores decreases, diffusion pathways become restricted and enzyme mobility decreases^50^. In wood-decay systems, for example, enzymes can advance ahead of the hyphal front only when sufficient moisture creates interconnected liquid domains^51^. Although enzyme transport has not been quantified specifically in conventional SSF substrates, a similar pattern is expected: connected aqueous domains allow enzyme diffusion, whereas dry or densely packed regions prevent it. As fermentation progresses, enzymatic degradation can locally soften the substrate and alter enzyme diffusion pathways, creating a dynamic interplay between substrate mechanics, enzyme mobility, and hyphal colonization.

### Toward designed substrate mechanics

Because substrate mechanics are rarely tuned in SSF, hyphal colonization and the depth of bioconversion are largely determined by the inherent mechanical properties of the raw materials rather than by intentional design. Future research should define measurable mechanical descriptors such as stiffness, softness, or viscoelasticity, and quantitatively relate them to colonization depth. Establishing such structure-function relationships will enable rational adjustment of substrate mechanics through particle packing, water content, or biopolymer concentration. In addition, the dynamic evolution of substrate mechanics during fermentation should be considered, as enzymatic degradation progressively alters material structure and influences fungal growth. Treating substrate mechanics as a design variable would enable deliberate regulation of colonization patterns and deeper bioconversion throughout the substrate.

## (3) Substrate architecture governs mass and heat transfer

**Fig. 4:**
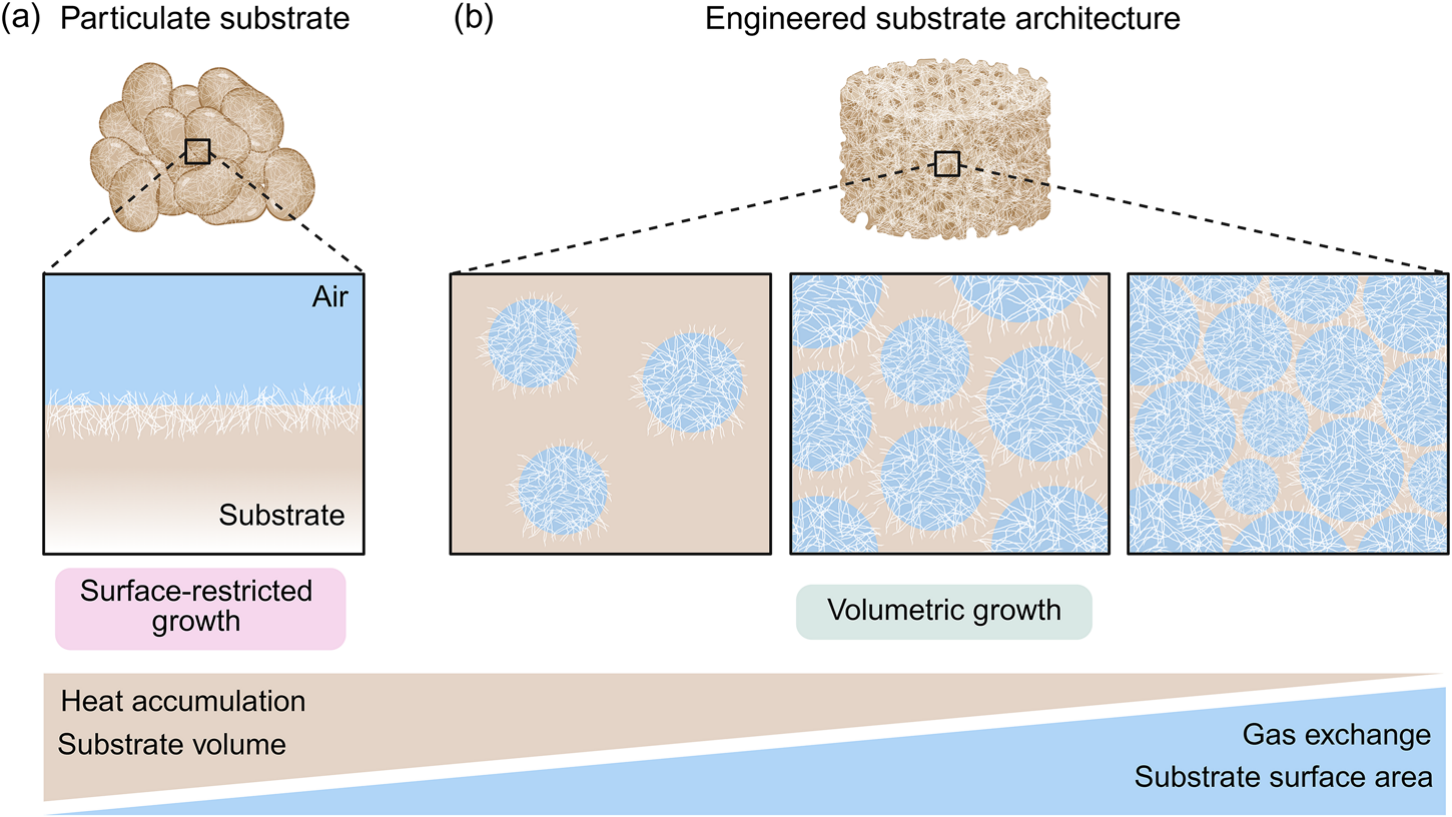
Substrate architecture governs mass and heat transfer during fungal solid-state fermentation. **a** Conventional particulate substrates have limited porosity and surface area, restricting fungal growth to the air-substrate interface. **b** Engineered substrate architectures with increased porosity and interfacial area improve gas exchange, heat dissipation, and nutrient accessibility, enabling volumetric fungal growth throughout the substrate. Figure was created with BioRender.

Volumetric and spatially uniform bioconversion in SSF largely depends on the mass and heat transfer properties of the fungi’s growth environment. Oxygen diffusion, nutrient accessibility, and heat dissipation depend on the spatial organization of pores and on how they are connected to one another. Substrate architecture is therefore a key design parameter for enabling volumetric fungal activity and bioconversion throughout the fermented material (Fig. 4).

### Limitations of conventional substrate materials

Most SSF processes rely on particulate substrates such as grains, legumes, or milling fractions (Fig. 4a)^12,52^. Their architecture is fixed by biological origin and random particle packing, resulting in limited porosity and low accessible surface area. Consequently, heterogeneous distribution of oxygen, nutrients, and metabolic heat develop within the substrate, confining fungal growth largely to the air-substrate interface and leaving deeper regions underutilized^12,53^. Efforts to mitigate these transport limitations have primarily focused on process interventions such as forced aeration, thin-layer configurations, intermittent mixing, and bioreactor innovations such as rotating drums, packed beds, or tray fermenters^6,52,54^. While these methods partially improve transport limitations, they do not modify the substrate’s architecture. Because mass and heat transfer directly depend on substrate structure, particulate substrates inherently limit volumetric colonization and the extent of bioconversion.

### Architectural parameters that govern mass and heat transfer

Transport phenomena within a substrate are determined by its porous structure, including porosity, pore size distribution, surface-to-volume ratio, and the extent to which pores form continuous transport pathways (pore connectivity)^55^. Increasing the surface-to-volume ratio increases the accessible area for fungal attachment and enzymatic activity, which can accelerate fungal colonization and nutrient bioconversion^56,57^. However, increasing porosity to achieve higher surface accessibility simultaneously reduces nutrient density, limiting the amount of substrate available to sustain metabolic activity^55^. This imposes a fundamental trade-off between surface accessibility and nutrient availability. An intermediate architecture would balance colonization area with nutrient availability, enabling uniform fungal growth and complete substrate utilization. Substrate architecture also affects the stability of the fermentation process by governing how effectively mass and heat can be exchanged throughout the substrate volume. Dense and low porosity substrates limit oxygen supply and CO_2_ removal, while porous structures facilitate gas exchange^58^. Pore structure similarly influences heat transport: fungal metabolism produces heat, and insufficient removal of metabolic heat can inhibit growth or inactivate enzymes^11,59^. In tempeh production, for example, increasing substrate bed depth has been shown to reduce fungal growth due to internal heat accumulation and oxygen limitation^60^. Intra-particle oxygen diffusion limitations persist despite external aeration, showing that process control cannot fully compensate for structural constraints of the substrate^61^. The importance of substrate architecture is also evident in blue-veined cheeses such as Gorgonzola, where deliberate piercing of the curd introduces air channels that enable internal oxygen transport and fungal colonization throughout the cheese matrix. Without these intentional pathways, fungal growth would remain largely surface-restricted. Substrate architecture, therefore, directly governs oxygen supply, heat dissipation, and metabolic activity throughout the substrate.

### Toward engineered substrate architecture

Because the architecture of particulate substrates is fixed, control over mass and heat transfer remains inherently limited. To support fungal metabolism throughout the entire substrate volume, materials with controllable architecture are needed (Fig. 4b). One approach is the use of engineered porous scaffolds^43^. Scaffold porosity, pore size distribution, and connectivity can be designed to meet the transport and metabolic requirements of the fungal system. Increasing porosity and interfacial area enhances gas exchange, heat removal, and fungal attachment, promoting continuous growth throughout the material. However, excessive porosity reduces nutrient density, imposing a trade-off between transport capacity and substrate availability. Future research should focus on identifying architectural descriptors that describe transport performance within substrates. Parameters such as total porosity, pore size distribution, and pore connectivity should be quantitatively linked to oxygen diffusion, heat dissipation, and spatial fungal colonization patterns. Establishing these structure-function relationships will be essential for rational substrate engineering. By decoupling mass and heat transfer from constraints imposed by raw materials, engineered substrate architectures enable more complete and volumetric bioconversion than conventional particulate substrates.

## Porous scaffolds as engineered substrates for solid-state fermentation

**Fig. 5:**
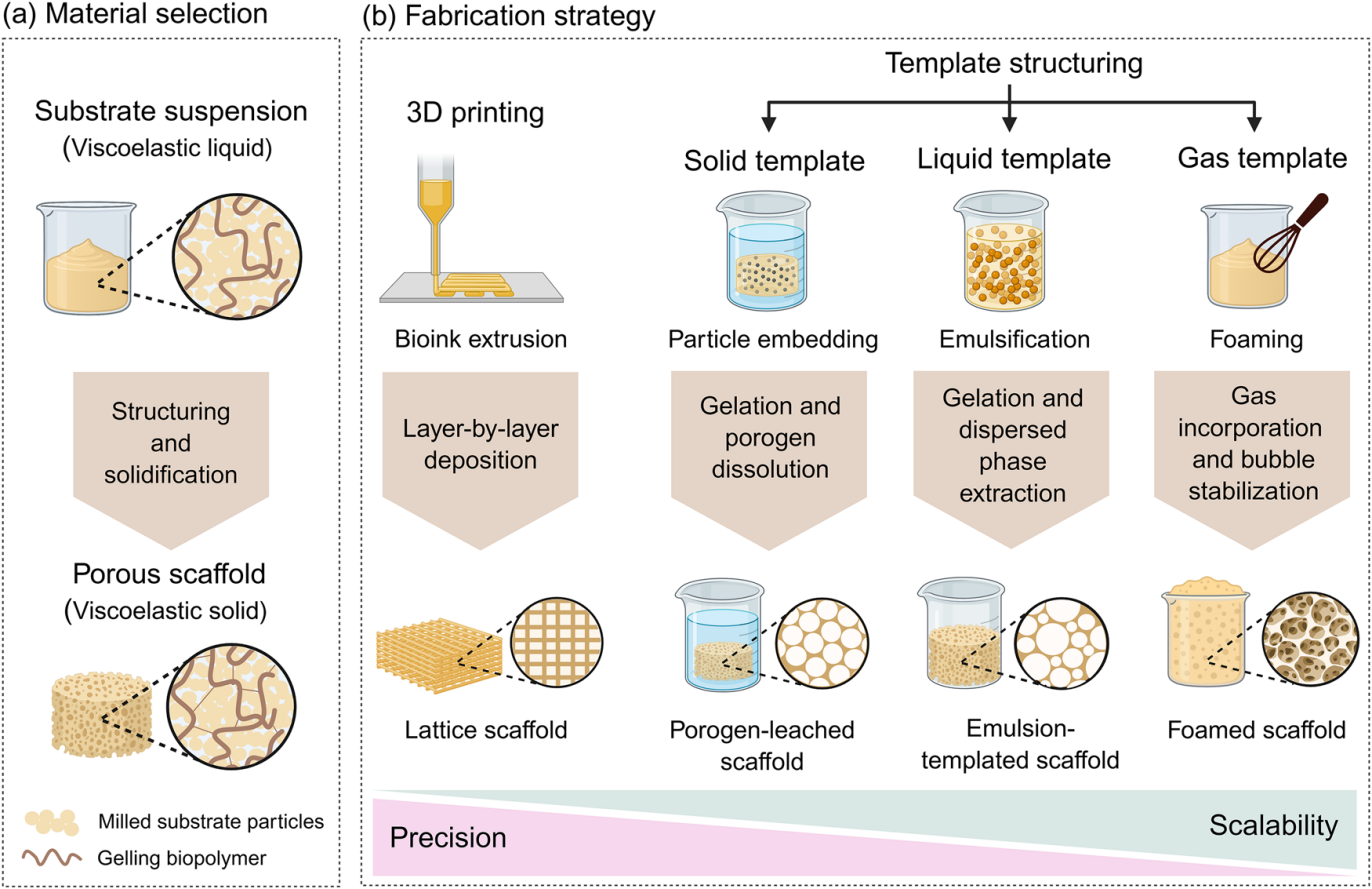
Material selection and fabrication strategies for porous scaffolds in fungal solid-state fermentation. **a** Substrate materials are formulated as concentrated viscoelastic suspensions that can be structured and subsequently solidified to lock in the desired architecture. **b** Porous scaffolds can be fabricated using complementary strategies with trade-offs between precision and scalability. Template-based methods such as porogen leaching, emulsion templating, and foaming offer higher scalability, whereas 3D printing provides greater control over scaffold architecture. Figure was created with BioRender.

Achieving volumetric and spatially uniform bioconversion in SSF requires substrates that integrate (1) biochemical affinity between fungal strain and substrate, (2) mechanical regulation of hyphal colonization, and (3) effective mass and heat transfer throughout the material. Conventional particulate substrates provide limited control over these properties. Engineered porous scaffolds enable rational design of substrate composition, mechanics, and architecture (Fig. 5), offering a platform for volumetric fungal colonization and uniform bioconversion. This section outlines key material considerations and fabrication strategies for constructing scaffolds for SSF.

### Material selection

Material choice defines biochemical affinity, mechanical properties, and processability into a porous structure (Fig. 5a). Scaffold materials must provide stable attachment sites for hyphae, withstand stresses imposed during colonization, and contain an internal pore network that ensures oxygen transport, heat removal, and metabolic activity. To be processed into a porous structure, materials must behave as liquids during structuring to allow pore templating and form self-supporting, solid-like structures during fermentation. These requirements can be met by using concentrated viscoelastic suspensions of milled substrate particles that can be structured and subsequently stabilized. Suspension properties, such as solid loading, particle size, pH, ionic strength, and the presence of gelling biopolymers, should be tuned to balance flowability during structuring with mechanical stability in the final scaffold. Food-grade gelling biopolymers and intrinsic gelling mechanisms of plant-based materials can be used to achieve this balance. Polysaccharides such as alginate, agar, carrageenan, guar gum, and pectin form gel networks at low concentrations and allow tuning of viscoelasticity and stiffness^62,63^. In addition, the intrinsic gelling properties of plant substrates can be exploited for scaffold stabilization: starch-rich substrates can be solidified through thermogelation^64^, pectin-rich substrates form gels via calcium crosslinking or sugar/acid-induced gelation^65,66^, and protein-rich substrates such as soy, pea, or lupin can be stabilized through heat- or pH-induced gelation^67–69^. Gluten-containing materials provide cohesive elastic networks formed by glutenin and gliadin to maintain mechanical integrity^70,71^.

### Fabrication strategies

Scaffold fabrication must process viscous food suspensions into porous structures with defined architecture. Two complementary fabrication strategies can achieve this: additive manufacturing (3D printing), which enables deterministic control over pore architecture, and template structuring, in which porosity forms through the removal or incorporation of a secondary phase (Fig. 5b). Extrusion-based 3D printing constructs scaffolds layer by layer with predefined pore size, spacing, and geometry^72^. This high level of control enables reproducibility and facilitates quantitative structure-function studies linking pore parameters to fungal colonization and bioconversion^45,73,74^. With recent advances in additive manufacturing, the use of food-grade and low-cost materials such as protein or polysaccharide bioinks has become increasingly more feasible^75^. However, the high level of precision comes at the expense of scalability, since current 3D printing methods operate in batch mode with limited throughput and relatively high processing costs^76^. Template-based approaches generate porosity by dissolving, removing, or expanding a secondary phase within the material. Methods such as porogen leaching^77^, emulsion templating^78^ and foaming^79^ create porous structures with high surface-to-volume ratios. While architectural precision is lower than in 3D printing, template structuring methods are more readily compatible with established large-scale food-processing operations. Their effectiveness depends on how suspension rheology and processing parameters interact to define the resulting scaffold structure.

### Toward scalable scaffold manufacturing

Together, material selection and fabrication strategies enable porous scaffolds that integrate biochemical strain-substrate affinity, mechanical regulation of growth, and controlled mass and heat transfer to support volumetric fungal activity. Implementing these design principles provides a route toward deeper and more uniform nutrient bioconversion in SSF. To translate these concepts into economically viable technologies, manufacturing approaches must be scalable. Future research should define quantitative relationships between processing conditions and the resulting mechanical and architectural properties of the scaffold. Particular attention should be given to manufacturing technologies that are compatible with existing food-processing infrastructure and continuous production formats. By linking material properties, processing parameters, and final scaffold performance, substrate engineering can move from laboratory-scale demonstrations to practical application.

## Conclusion and outlook

This perspective reframes the substrate as an active process component in SSF. By integrating (1) biochemical strain-substrate affinity, (2) mechanical regulation of hyphal growth, and (3) architectural control over mass and heat transfer, substrate properties become directly linked to fungal metabolism and the resulting bioconversion. Rather than optimizing strain performance and process conditions alone, this approach extends process control to the substrate, enabling better control over where and how fungal growth and bioconversion occur within the material.

Implementing these principles in practice requires systematic, quantitative approaches that connect fungal metabolism to measurable material descriptors and scalable manufacturing strategies. The development of high-throughput characterization methods combined with data-driven strain selection will enable rational strain-substrate pairing based on biochemical affinity. Establishing quantitative relationships among substrate mechanics, architecture, and fungal colonization patterns will guide scaffold design. In parallel, scalable manufacturing approaches will be needed to translate substrate engineering into economically viable technologies.

Beyond nutritional improvement, volumetric fungal growth throughout the substrate can also influence the texture and structure of fermented foods. When fungal biomass develops not only at the surface but throughout the material, it modifies the internal architecture, affecting properties such as firmness, cohesiveness, and structural stability. The same principle extends beyond food texture: volumetric mycelial growth can also be used to engineer composite materials with defined mechanical and functional characteristics. By regulating where and how fungal biomass forms within a substrate, it becomes possible to design both sensory properties in foods and structural performance in mycelium-based materials.

By treating the substrate as an active process component rather than a fixed constraint, SSF can move from empirically optimized formulations toward intentionally designed systems. This approach positions SSF as a versatile and scalable technology for developing next-generation fermented foods and fungal-based materials.

## Data Availability

No datasets were generated or analyzed during the current study.
